# Physical Activity and Body Image Perception in Adolescents: A Systematic Review

**DOI:** 10.3390/ijerph192013190

**Published:** 2022-10-13

**Authors:** Emanuela Gualdi-Russo, Natascia Rinaldo, Luciana Zaccagni

**Affiliations:** Department of Neuroscience and Rehabilitation, Faculty of Medicine, Pharmacy and Prevention, University of Ferrara, 44121 Ferrara, Italy

**Keywords:** adolescence, body image, body dissatisfaction, physical activity, body mass index

## Abstract

Adolescence represents a vulnerable phase of life for psychological health. The practice of physical activity (PA) appears to have a positive influence on adolescents, increasing self-esteem and producing a more positive body image. A systematic review of published articles over the past 10 years until June 2022 was conducted according to the PRISMA statement employing the electronic databases MEDLINE and Web of Science (639 records) to summarize the literature on the relationship between body image dissatisfaction (BID) and assessed by figural scales and practice of structured and unstructured PA in adolescents (10–18 years), taking into account BMI and/or weight status. All articles were independently reviewed using inclusion/exclusion criteria, retrieved data, and assessed quality with the adapted Newcastle-Ottawa Scale for observational studies. The main finding of interest that emerged from most of the 28 included studies is the negative association between BID and PA during adolescence: as PA increases, BID decreases. However, this updated systematic review also identified some flaws in the existing literature, highlighting the need for high-quality adolescent research using validated figural scales and objective PA assessments. In conclusion, the reviewed studies showed that PA involvement can be efficacious in protecting from body image perception concerns and enhancing body satisfaction. Future interventions should promote structured and unstructured PA during adolescence to improve self-esteem and body image.

## 1. Introduction

Overweight/obesity is a public health problem constituting an independent risk factor for several chronic non-communicable diseases and increased mortality [1,2]. Globally, there has been an increase in obesity and a decline in the underweight: the increase in obesity, including severe obesity, may be linked to increasing social disparities that limit healthy food access for people at the greatest risk of malnutrition [3]. According to WHO [4], the worldwide obesity prevalence nearly increased three-fold between 1975 and 2016, and more than 340 million children and adolescents (ages 5 to 19) were overweight or obese in 2016. During the recent COVID-19 pandemic, the increase in the rate of body mass index (BMI) almost doubled in comparison with the pre-pandemic period in persons aged 2–19 years [5]. 

There is scientific evidence that an unhealthy body image (BI) is linked to obesity and physical inactivity, exerting a key role in the emergence of eating disorders during adolescence. The image we have of our bodies is a dynamic feature that can change over a lifetime but the stage of adolescence is crucial for the development of a healthy BI due to the age-related transitions that occur during this period [6]. Sociocultural context greatly influences how adolescents perceive their bodies. The influence of body ideals interacts with the critical period of adolescence characterized by fast and diversified physical modifications that include changes in primary and secondary sexual characteristics, body shape, size, and composition. Key influences on BI include media, which target adolescents, and peers, which contribute to perceived body ideal beliefs. Especially the mass media can lead adolescents to internalize Western society’s ideals of physical attractiveness and beauty, leading to body dissatisfaction when these standards are perceived as not being met [7,8]. Recent findings have also shown that greater use of social media (Instagram and Snapchat) is associated with higher body dissatisfaction [9]. In accordance with sociocultural models of the development of body dissatisfaction and eating disorders [10], pressures on appearance from peers, family, and the media, and psychological processes participate in the onset and persistence of body dissatisfaction: internalization and pressures to conform to these socially prescribed body ideals explain the associations between weight status and BI [11].

Following Sirard and Pate [12], physical activity (PA) can be identified as any type of body motion generated by skeletal musculature that causes an expenditure of energy. Physical inactivity is one of the possible negative health consequences that may result from the interaction between weight status and BI. However, this consequence is controversial: overweight/obese individuals tend to avoid PA according to some studies [13], while others found no significant differences in reaching the recommended level of PA based on weight status [14]. PA is a health-promoting behavior, although it should be pointed out that compulsive exercise is a commonly used strategy to offset caloric intake or reduce body weight. A recent review [10] found that a positive BI is associated with greater participation in PA and sports, in contrast to a negative BI. The association between PA and BI is complex: while, on the one hand, PA practice results in perceptible physical changes (weight, body composition, etc.), thus contributing to self-confidence and consequently leading to improved BI, on the other hand, BI may discourage or motivate PA and sports participation [15]. Furthermore, sex affects the association between PA and BI differently, with girls showing a more complex relationship [16]. The role of sex in the relationship between BI and PA is still not fully elucidated probably due to possible differences between the two genders “in moderating variables that impact the relationship between PA and body image” [17]. The same notion of femininity complicates this relationship, driving girls to worry about their appearance when they are engaged in PA. In confirmation of this, a recent study showed a positive association between MVPA and self-esteem among boys only [18].

Previous studies have particularly emphasized BI in the female sex, due to its greater dissatisfaction and the prevalent desire to be thinner with significant differences between actual and ideal silhouettes [19]. The pattern in the two genders is indeed different with higher female body image dissatisfaction (BID), indicating the greater importance given by females to physical appearance compared to males [19]. The relationship between body dissatisfaction and BMI was found to be linear in females [20]. In males, less dissatisfaction has been evidenced, although this concerns both the overweight (wanting to be thinner) and the underweight (wanting to be larger): the relationship between BID and BMI is curvilinear [21].

A major limitation of some previous syntheses is to compare BI assessed by different methodologies (25 different measures of BI were found in the studies reviewed by the synthesis of Hausenblas and Downs [22]), including questionnaires, surveys, and figural scales. A recent systematic review [23] pointed out that figural scales are the most widely adopted visual tools for assessing BI and body perception, by measuring the discrepancy between perceived and ideal BI; they are quick, convenient, and inexpensive to administer. 

Given the controversial findings in the literature and some limitations of the currently available syntheses, we thought it might be of major interest to explore the relationships between PA and BI assessed by figural stimuli, taking into account sex and weight status or BMI during adolescence, a phase of great physical and psychological changes and characterized by a strong reduction/abandonment of previously undertaken sports activities. Due to the lack of explicit theory to guide the study of BI and PA, within the framework usually employed [17,24,25], we considered sex, age, BI assessed by figural stimuli, and PA characteristics as specific key variables. In addition, BMI was considered as an additional variable related to individual characteristics. Therefore, the aims of this review are: i. to evaluate the associations between BI or BID and PA according to BMI or weight status in adolescents; ii. to allow a more complete and up-to-date synthesis of the studies that have been carried out in this area by figural scales over the past decade; iii. to extend the analysis on BI or BID to PA contexts related to lifestyle -not just structured PA. In addition, finding possible gaps in the existing literature can direct future research on specific aspects of the relationship between PA and BID.

## 2. Materials and Methods

This review was carried out according to the PRISMA guidelines (Preferred Reporting Items for Systematic Reviews and Meta-Analyses) [26]. The PRISMA checklist was reported in Supplementary Materials (Table S1). The protocol of this review was registered with PROSPERO (CRD42022342891).

### 2.1. Search Strategy and Selection Criteria

The systematic search was conducted in the PubMed and Web of Science databases on articles published in the last ten years until 13 June 2022 by the following combination of search terms: (“body image” or “body dissatisfaction” or “body satisfaction” or “body representation” or “silhouette scale”) and (anthropometry or BMI or weight or “fat mass” or adiposity or “body composition”) and (“sport activity” or “physical activity”) and adolescen*. No geographical or cultural limitations were applied. The topic analysis was first discussed and agreed upon by all three reviewers. Articles resulting from database searches were then imported into Covidence software (Veritas Health Innovation Ltd, Melbourne, Australia), where duplicate records were removed. The search was then continued manually and independently by two reviewers (L.Z. and N.R.), discussing with the third reviewer (E.G.-R.) in case of discrepancies. Further eligible studies were included through consultation of the reference lists of previously selected articles.

All quantitative studies that investigated the possible association between BI or BID and PA, taking body composition into account, were included. Editorials and commentaries, case studies, protocol studies, conference proceedings, books and book chapters, and theses were excluded. In addition, excluded were studies related to the development and/or validation of BI screening tools, and studies in which BI assessment focused on specific features (e.g., muscularity) or parts of the body (e.g., breasts). We detailed the selection criteria used in Table 1.

The relevant data were extracted from the selected articles as follows: article authors and year of publication; study design; geographical location; race/ethnicity; sample size; sex; age; BMI and/or weight status; PA assessment; silhouette rating scale used; BID and/or satisfaction; main outcomes according to the aims of this study. Due to the lack of quantitative homogeneity in the reviewed studies, we could not conduct a meta-analysis. The findings are therefore presented as a narrative analysis: all studies selected from the database searches were summarized in tabular form and organized in alphabetical order. 

### 2.2. Quality Assessment

The quality assessment of selected studies was performed according to the adapted Newcastle-Ottawa Scale (NOS) [27] for observational studies. NOS is based on (1) clarity of the study objective, (2) sample selection (representativeness of the sample; sample size; response rate; ascertainment of exposure), (3) comparability (control of confounding factors; comparability of participants from different outcome groups), (4) outcome (assessment; statistical tests). Ascertainment of exposure (within criterion 2) was made with reference to the figural scale used (validated or not validated). The overall score for each study (range 0–16) is derived from the ratings of the single reported components. Based on Hillen et al. [27], studies that received 13 to 16 out of 16 possible points (scores > 75%) were considered of high quality, 9 to 12 points of moderate quality (scores > 50%), 8 and below of low quality (scores ≤ 50%). A low overall score is indicative of a higher risk of bias in the study. This assessment was independently carried out by two reviewers (N.R and E.G.-R.). Any disagreements between the two reviewers were discussed and resolved with the assistance of the third reviewer (L.Z.).

## 3. Results

### 3.1. Study Selection and Description

**Figure 1:**
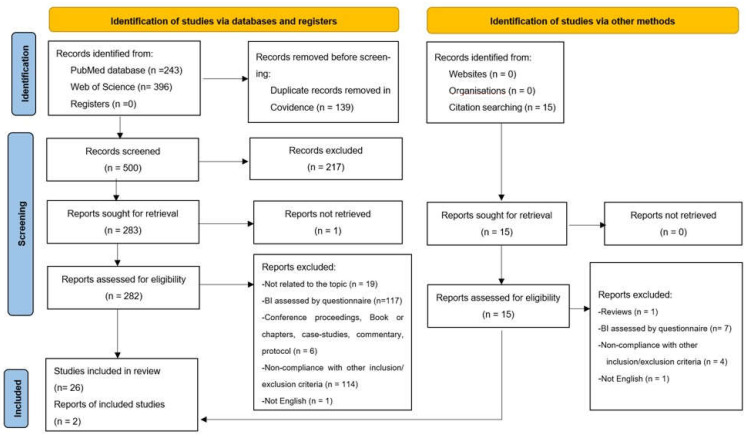
Flow chart of the study selection process (PRISMA 2020 flow diagram).

A total of 639 articles were identified through the search conducted on the databases. Following the article selection procedure (Figure 1), 28 studies were included in the systematic review. After removing 139 duplicates, 500 articles were screened against title and abstract for eligibility for inclusion criteria. Studies excluded after the title and abstract screening were 217. Therefore, 283 studies were assessed for full-text eligibility; however, one study could not be retrieved. Thus, 282 studies were the number of articles assessed for full-text eligibility. Of these, 257 were excluded for several reasons: BI assessed by questionnaire (n = 117); conference proceedings, book or book chapters, case studies, commentary, protocol (n = 6); not written in English language (n = 1); non-compliance with other inclusion/exclusion criteria (n = 114); not related to the topic (n = 19). Therefore, 26 articles met the inclusion criteria. Two additional articles were included in the review from citation searching, so the articles included in the review were 28. 

### 3.2. Characteristics of Studies

Twenty-eight studies were included in this review (Table 2): 13 of which were carried out in Europe (7 in Spain, 4 in Italy, and 1 each in Finland and Portugal), 8 in Asia (2 in Malaysia, 2 in China, and 1 each in Thailand, Indonesia, Kuwait, and Israel), 3 in North America (all in the USA), 3 in South America (all in Brazil), and 1 in Africa (in Ghana). 

The number of subjects surveyed in the studies ranged from a minimum of 36 subjects (a study on selected athletes—male soccer players) to a maximum of 10,496 Finnish adolescents. In particular, looking at the number of subjects examined, only 1 study (3.6%) had fewer than 100 subjects, 14 studies (50.0%) had fewer than 1000 subjects, 11 studies (39.3%) had fewer than 5000 subjects, and only two studies (7.1%) had more than 5000 subjects. Studies with the smallest sample sizes preferably involved only one sex and longitudinal or repeated measures design. Most samples included both genders, 5 studies (17.8%) included only females, and one (3.6%) only males. All the studies were conducted on samples of students except two studies on athletes, two studies on Chinese samples [46,69], that employed a secondary-analyses of data from the China Health and Nutrition Survey (CHNS), and the Indonesian household survey [59]. The majority of studies had a cross-sectional design, and only three studies had a longitudinal or repeated measures design [38,42,63].

For what concerns BMI and weight status, 12 studies (42.9%) reported the percentage of weight status categories (the majority determined from the cut-off proposed by Cole et al. [70,71] adjusted by age and sex, and two by the BMI percentiles (Underweight: <5th BMI-P; Normal weight: 5th to 85th; Overweight: 85th to 95th; Obese: ≥95th), while two others used Z-score of BMI for age (BMIZ) (thinness was defined as BMIZ less than −2 and overweight or obesity above 1), 7 (25%) reported mean values of BMI, and nine (32.1%) reported both mean values of BMI and the percentages of weight status categories.

The mean BMI of the participants included in the samples retrieved in the present review ranged from 16.9 kg/m^2^ of the youngest female sample of rhythmic gymnasts aged 10.7 years [53] to 22.5 kg/m^2^ of Spanish male sedentary students aged 12–17 years [50]. The female adolescents of Kuwait reported the highest percentage of OW/obesity (61.4%), of which 44.3% of obese. In addition, the USA samples reported a high percentage of overweight/obesity: 50.7% in the girls [45], of which 31.6% of obese, and 45% in the mixed sample in the longitudinal study of Schuster et al. (survey 1) [38]. The lowest percentage of OW/obese was reported in the Indonesian sample [59], with 14% of OW/obese for boys and 11% OW/obese for girls, but a high percentage of stunting and thinness (19% and 10% in males; 23% and 4% in females, respectively) was also reported. Africans [44] and Israelians [35] showed a low percentage of OW/obese (19.5% in the former, 20.4% (for males), and 16.9% (for females) in the latter).

PA was assessed with different instruments: the majority with indirect methods such as questionnaires, validated (IPAQ, PAQ, 24 h Physical Activity Recall, Baecke questionnaire) or not validated (a single question on h/week of sports training or questions on PA) to divide the samples in active and sedentary or in categories according to PA levels (low, moderate or high PA). Only three studies have directly assessed PA: one by the pedometer, the other by the pedometer plus the 24 h PA recall, and the last one by the accelerometer to determine the sedentary time, light PA, moderate PA, and vigorous PA (min/h) plus a self-reported questionnaire. In epidemiological studies, self-reporting is usually the most feasible method of assessing PA, especially when a high number of participants are interviewed due to the relative inexpensiveness, the minimal inconvenience to the participant, and the relative ease of administration. 

According to the inclusion criteria, we chose only studies performed using the body silhouette scales for the assessment of BI and BID. Eleven studies assessed BI using the Stunkard Rating Scale with nine silhouettes, six studies used the seven silhouettes developed by Collins for preadolescents, three studies used the Contour Drawing Rating Scale proposed by Thompson and Gray, three studies on Brazilian samples used the silhouettes developed by Kakeshita et al. [48], one study each the BIDA questionnaire, the Body Silhouette Chart developed by Sanchez-Villegas et al., and the 13 silhouettes developed by Gardner et al. adapted for Spanish by Rodriguez et al. [52]. Zach et al. [35] used five silhouettes (not reported) drawn by an artist, and Min et al. [46] used nine new silhouettes reported in the paper. From the current (also called actual or feel) and ideal figures, the researchers calculated some indices of dissatisfaction, which, under different names, generally assessed the discrepancy between feel and ideal BI, thus coming to define the percentage of satisfied/dissatisfied (without discrepancy between current and ideal figures or with the discrepancy, respectively) subjects or mean value of dissatisfaction (negative values mean a desire of a bigger body and positive values mean a desire of a thinner body). 

### 3.3. Association between Weight Status, PA, and Body Dissatisfaction

Several studies underlined the association between BI and BMI, especially in overweight and obesity categories [38,41,43,46,57,59,60,63,65,67]. In almost all the studies that reported this association, overweight and obese adolescents had higher body dissatisfaction and less body perception consistency, evaluating themselves as slimmer than they were. In contrast, Zach et al. [35] reported that a large percentage of overweight and obese adolescents were satisfied with their body shape and size, regardless of sex. Moreover, Michels et al. [44] did not find any significant association between the level of body dissatisfaction and BMI. 

Ten studies [31,33,36,38,46,63,65,66,67,69], despite evaluating both body dissatisfaction and PA or sports levels, did not assess the association between these variables.

Six studies that analyzed the association between PA and BID found no significant association or correlation between these two variables [28,35,43,44,54,55], and nine studies reported a negative association between PA and BID or body perception [39,41,42,45,53,57,58,62,68], whereas one study reported an increase in body dissatisfaction with an increase in PA level [47].

### 3.4. Quality of Studies

Half of the examined studies showed a high level of quality with an overall score ≥ 13 (Table 2), and the remaining 14 were found to be studies of moderate quality (8 < score < 13). No study received an overall score of <9 (Table 2, Table S2). 

In more detail, 15 studies (53.6%) provided an adequate description of the random sampling process, 16 studies (57%) provided an adequate description of the study sample, 25 studies (89.3%) used a validated measurement of BI, and 21 studies (75%) controlled for potential confounders.

## 4. Discussion

The principal aim of this systematic review was to investigate the association between body image dissatisfaction (BI and BID) and PA practice in adolescents, taking BMI or weight status into account. The hurdles we addressed primarily involved the assessment of dissatisfaction with BI, which was reported as an index of the discrepancy between the actual and ideal figure in some studies, while others reported the percentage of satisfaction or dissatisfaction. The type of BI rating scale used differed in the studies reviewed, although the use of Stunkard’s scale [29] prevailed. Second, we found a variety of methods for assessing PA in the studies reviewed. Because of the different measures of BI and BID, and PA, a narrative review of the 28 included studies was conducted. 

Despite the difficulties faced, this review provides an important update of the literature related to PA and BID in adolescents over the past decade, highlighting a slightly higher interest in the female sex (5 out of 28 studies; while 1 out of 28 studies involved only the male sex and 4 were mixed-sex studies). Although the limited number of reviewed studies on adolescents and the different survey methodologies applied to preclude a decisive view of the relationship between BI/BID and PA, the main findings of this systematic review indicate mostly (56.3% of studies) a negative association (moderate-medium) between PA and BID in adolescents, coming to highlight a better body perception in the physically active adolescents [50] and an association between BI disorders and reduced moderate-to-vigorous physical activity (MVPA) [58]. However, due to differences among samples in PA practiced (amount and type of activity), weight status, and sex of participants, this relationship is complex, and the results contradictory, so much so that 37.5% of reviewed studies detected no association between these variables and 6.3% showed a positive association. In contrast, generally, higher body satisfaction results in active adolescents or those with high PA levels, and lower body dissatisfaction in sedentary or physically inactive girls was also reported [47]. In the field of sports, Fernandez-Bustos et al. [50] found greater dissatisfaction in girls who practiced aesthetic/light PA or were inactive than in those who practiced other types of PA, confirming the importance of the type of PA practiced. Furthermore, Zaccagni et al. [53], analyzing a sample of rhythmic gymnasts, pointed to a possible source of misunderstanding in the ideal BI, showing that the ideal figure in sport does not coincide with the ideal figure in everyday life—the dissatisfaction between perceived and sport ideal figure was greater than the dissatisfaction between perceived and general ideal figure.

Diverse physical, cultural, social, and psychological changes that characterize early and medium adolescence interact with BI and BID between ages 10 and 18. Restricting to physical changes during this period, sex-specific differences occur that result in an increase in body fat mass and a decrease in lean mass in the female sex compared to the male, with consequent changes in body shape and size. The eventual practice of PA and sports may also interact, leading to eventual improvements in the perception of BI [15]. However, a decline in PA practice during the period of adolescence is well known [72,73], leading especially girls to be less active than boys and not following the WHO recommendations of at least 60 min of MVPA per day [74], as confirmed by this review with a percentage of inactivity reaching almost three times as high in females as in males in Spain [50] and exceeding 80% inactivity in Brazilian girls [47] with less than 10% of girls participating in the recommended 60 min/day of MVPA for seven days in the USA [45]. Inactivity in China was high (about 60%) but similar in the two sexes [69], while in Indonesia, girls met the daily PA recommendations slightly less than boys [59]. The only exception is the high MVPA averages (over 5 h per day, value not disaggregated by sex) reported for Ghanaian adolescents [44]. 

Because body weight dissatisfaction is reported as a factor associated with lack of PA, physical inactivity can result, as a consequence, of the interaction between weight status and BI, according to Duarte et al. [73]. An alternative hypothesis is that, instead of discouraging, BI may motivate PA and sports participation [15]. In general, adolescent girls of all ethnic groups showed greater body dissatisfaction than boys. However, in addition to Malaysia, dissatisfaction appears lower in adolescents from African countries and African Americans than in adolescents from Western countries, in agreement with the literature [75]. In Ghanaian adolescents, in particular, the greatest body dissatisfaction involved a body that was too thin [44], unlike the other studies reviewed. This result is consistent with the known tendency of non-White populations (particularly Africans and African Americans) toward a more favorable BI than Whites and a preference for a heavier body [25,76] according to a different beauty ideal. Despite the greater dissatisfaction that generally characterizes Western countries, Finnish adolescents show a high percentage of satisfaction with their BI (about 60%), with small differences between genders [68]. This pattern may at least partly be explained by a possible larger presence of preadolescents in the sample characterized by a lower age range (9–12 years) than in most of the studies considered. Indeed, it is believed that the physical, cultural, and diminished self-esteem changes that occur during adolescence can contribute to an increased awareness of BI, resulting in greater concerns about weight (particularly in females) and musculature (in males) [77,78].

Other previous analyses conducted on adults had shown small/medium associations between PA and BI [17,25], pointing to a stronger PA impact of PA on BID among young people, according to Bassett-Gunter et al. [17]. In particular, the number of hours spent in sports activities was found to be a predictor of body dissatisfaction with a decrease in BID as the number of hours spent in sports activities increases [19]. In this review, a positive effect of sports training on BID was found in a study [42] carried out by a repeated measure design: a decrease in BID and changes in anthropometric measurements were evident after 12 weeks of soccer training. 

An important aspect of the relationship between PA and BI/BID may be the intensity of PA. Although it is plausible that this factor, as well as multiple others, is involved in the association, it is not possible to explore it in depth because of the limited data provided by the studies reviewed and the heterogeneity in the methods used to assess PA (from different types of questionnaires to accelerometers). Taking into account the accuracy and reliability of accelerometry [79], we emphasize the need for adequate studies designed to allow an understanding of the actual role of PA on BI/BID. According to Gualdi-Russo et al. [80], a decrease in body adiposity occurs in children and adolescents as MVPA increases and sedentary time decreases. If PA affects adiposity and, in particular, weight status, this could reverberate on BI/BID.

Several anthropometric parameters (BMI, waist circumference, waist-to-hip ratio, waist-to-height ratio) are possible indicators of overall and central obesity and predictors of weight status [81,82]. However, the vast majority of examined studies have only considered BMI, classifying adolescents into weight categories. According to NCD-RisC [83], BMI can be regarded as “an imperfect measure of the extent and distribution of fat in the body, but it has the major advantage of having consistent and comparable data in many population-based surveys”. The relationship between weight status and BI/BID is complicated and could significantly affect the health behaviors of adolescents. Specifically, negative health consequences that may result from the interaction between weight status and BI include eating disorders, physical inactivity, and dysfunctional exercise [6]. Adolescent girls, in particular, will be at higher risk of developing clinical eating disorders because of intense BID and peer pressure to look thinner. At the same time, although PA is usually encouraged as a behavior that promotes health [79,84], compulsive exercise is a commonly used purging strategy to balance caloric intake or modify weight, body size, or shape [85]. This is probably the source of some apparent inconsistencies in the examined literature indicating greater PA practice in overweight/obese adolescents or those who perceived themselves as fat [35], although some examined studies show little awareness of the importance of PA among overweight/obese adolescents [46,59]. Lastly, further inconsistencies in the analysis of relationships between BID and BMI may arise from the study of sex-mixed samples (n = 4) due to the different characteristic patterns of the two genders (linear relationship in females, curvilinear relationship in males).

### Strengths, Limitations, and Directions for the Future 

This systematic review provided an overview of the literature of the last 10 years and identified eventual gaps in the literature to improve future studies. Among the strengths of this review is the systematic search of articles from the literature by evaluating a wide variety of evidence. A specific strength relates to including only studies that have assessed BID through figure scales. A limitation of this review is that we screened only two major databases, and we purposely decided to exclude gray literature. Moreover, other limitations reflect the limitations of the studies analyzed. First, we chose to consider only studies that used figural stimuli, but there is evident heterogeneity in the figural scales used, as well as in the methods employed to assess PA. With particular reference to BI, some studies have relied on unclearly defined or non-validated body image rating scales or inappropriate BI rating scales (e.g., scales developed for preadolescents or adults but applied to adolescents) with results of weak or unknown value. The use of small convenience samples in several studies is an additional limiting factor in assessing an association between BID and PA. Concerning PA, the limited information on this variable did not allow us to detect any abnormal compulsive exercise behavior. Accurate measurements of the PA level would be needed to assess the real impact on BI. In future studies, we suggest further investigation of the quality and quantity of exercise performed to discriminate behaviors attributable to dysfunctional exercise. The same assessment of overall and central obesity, limited in this review to BMI because it was found to be the most widely used, was obtained from directly measured anthropometric measurements in some studies and in other studies from self-reported height and weight values resulting in a possibly incorrect estimate. Secondly, the studies reviewed were generally cross-sectional, the limit of which is often ascribed to the impossibility of establishing causal relations. However, Wunsch et al. [86] think that this possibility is rather related to “whether or not our modeling strategy is structural”. Therefore, more robust study designs and/or modeling strategies should be employed in the future to facilitate the understanding of causal relations between PA and BI/BID. Given the significant differences between genders in BID (dissatisfaction more pronounced with a clear preference toward slimmer figures in girls) and PA (lower amount in girls that are also less interested in vigorous PA), it is recommended that statistical analyses be conducted separately by reporting data by sex. Moreover, there is a need for future research to improve and standardize the methodologies used, especially in PA and BI assessments.

Finally, the exclusion of non-English articles certainly resulted in a loss of information, but we felt this choice was in line with current international systematic syntheses. 

## 5. Conclusions

Up-to-date local data on adolescents’ BI perception and PA practice are indispensable for planning health strategies and supporting non-communicable disease management. Monitoring changes in ideal BI during adolescence should be a primary health objective to guarantee the psychological and physical well-being of adolescents: health interventions should include strategies to promote health-related benefits and positive BI through PA among adolescents. The results highlighted in this review show a ubiquitous prevalence of unsatisfactory BI, although more pronounced in Western countries, with a concurrent trend toward inactivity and overweight in adolescents. This picture implies a high risk of the spread of nutritional disorders. Considering social and behavioral differences among subgroups based on sex, BMI, and PA, a unique approach may be inadequate: specific recommendations tailored to the needs of adolescents should be provided by researchers and health professionals. 

## Figures and Tables

**Table 1:** Inclusion and exclusion criteria used in the articles screening.

Inclusion Criteria	Exclusion Criteria
-Original article in English	-Assessment of body dissatisfaction only by questionnaires
-Full-text availability	-Assessment of satisfaction/dissatisfaction with specific body features or parts
-Adolescents aged 10–18 years or mean age within this range	-Development or validation of new BI screening tools
-Assessment of body image perception	- Studies of individuals affected by pathologies
-Use of body silhouettes scale for the assessment of BI and BID	-Duplicate studies * or studies with partially overlapping samples
-Assessment of PA	-Literature reviews
-Assessment of indicators of overall and central obesity (at least BMI)	-Editorials, commentaries, case and protocol studies, conference proceedings, books and book chapters, theses

* In the case of additional data on the same sample, both studies were cited.

**Table 2:** Literature studies reported in chronological order on body image dissatisfaction, PA, anthropometric profile in adolescents.

Study Reference -Design-	Country/Ethnicity	Sex: Number of Subjects	Age (Years)	BMI (kg/m^2^) and/or Weight Status	PA (Tool)	Body Dissatisfaction	Figure Rating Scale	Main Findings	Study Quality (Score)
Chen and Wang, 2011 [28] -cross-sectional-	Chicago, USA low-income African Americans	M + F: 402 (49% F)	10–14	*Weight status* OW/obesity M: 3 3%; F: 42% M + F: 37%	Mean values not reported (questions)	*Mean Ideal BI* M: 4.19 ± 0.79 F: 4.20 ± 1.05 Body size perception: Among OW and obese adolescents, 69% thought they were OW or obese (boys: 54%; girls: 78%) (only ideal body image (IBI) was assessed)	8 silhouettes adapted by Stunkard et al. [29]; Stevens et al. [30]	Girls with smaller IBI were more likely to participate in PA less than 3 days/week. No significant association was found between IBI and PA in males.	9
Bibiloni et al., 2012 [31] -cross-sectional-	Balearic Islands	M: 939 F: 1022	12–17	*Mean BMI by PA behavior* M: Sedentary: 22.5 ± 3.8 Active: 21.7 ± 3.8 F: Sedentary: 21.6 ± 4.0 Active: 22.3 ± 3.8 (sedentary cut-off level = 300 min of MVPA per week.)	*PA categories* M Inactive: 22% Active: 78% F Inactive: 51% Active: 49% *PA practice* M *Moderate* None: 11% Mean duration: 215.1 ± 250.5 min/week *Vigorous* None: 11% Mean duration: 502.8 ± 401.4 min/week F *Moderate* None: 13% Mean duration: 169.1 ±165.7 min/week *Vigorous* None: 28% Mean duration: 354.3 ± 356.2 min/week (IPAQ-A)	*Desire (%) for weight change in sedentary adolescents:* M -want to be thinner: 31% -want to remain the same weight: 13% -want to be thicker: 23% F -want to be thinner: 51% -want to remain the same weight: 50% -want to be thicker: 54%	9 silhouettes developed by Stunkard and Stellar [32]	Age, sex, and dissatisfaction with body size, in addition to other components, are important factors for PA practice. Association of BI or BID with PA or BMI or weight status not assessed.	15
Petracci et al., 2013 [33] -cross-sectional-	Emilia-Romagna region, Italy	M: 2181 F: 2157 M + F: 4388	*Mean* 13.8 ± 0.4	*Weight status* Not OW: 75% OW: 20% Obese: 5%	*PA practice* 44%: <2 h/w 42%: 2–11 h/w 10%: ≥11 h/w (PA measured using only one question: hours per week)	*BID* 37% satisfied (FID = 0) 14% want to be larger (FID < 0) 43% want to be thinner (FID > 0)	9 silhouettes developed by Collins [34] (an incorrect number of figures seems reported: 9 instead of 7)	62% of the children reported accepting their physical appearance but with a tendency to indicate a heavier self-figure than their ideal one and having an inadequate BI perception relative to their BMI. Association of BI or BID with PA or BMI or weight status not assessed.	15
Zach et al., 2013 [35] -cross-sectional-	Israel Jewish, Arab, Bedouin and Druze	M: 2562 F: 3085	*Mean* M: 15.04 ± 1.68 F: 15.20 ± 1.60	*Weight status* M UW: 5% NW: 74% OW: 13% Obese: 7% F UW: 4% NW: 79% OW: 13% Obese: 4%	*PA level* M Inactive: 29% Insufficiently active: 53% Sufficiently active: 18% F Inactive:42% Insufficiently active: 54% Sufficiently active: 5% (PA level by questionnaire: any moderate and/or vigorous level of PA performed for 60 min/day = sufficient; lesser amount = insufficient; no activity or activity less than once/week = inactive. PA intensity by questionnaire: based on the perceived difficulty of exercise as light, moderate and vigorous by the Borg perceived exertion scale)	*Body satisfaction* M OK: 77% Too fat: 12% Too thin: 10% F OK: 73% Too fat: 23% Too thin: 4% *Satisfaction of body shape and size in OW, obese* M OW: 66% Obese: 41% F OW: 47% Obese: 26% *Body perception* M Very fat: 2%; Fat: 13%; Average: 45%; Thin: 34%; Very thin: 5%. F Very fat: 2%; Fat: 15%; Average: 37%; Thin: 37%; Very thin: 10%. (Body perception by the chosen number of the current figure inside the scale where 1 represents an obese figure and 5 a lean figure. Body satisfaction by questionnaire: I am OK, too fat or too thin)	5 silhouettes drawn by an artist (not reported)	A large percentage of OW/obese and more than half of UW adolescents were satisfied with their body shape and size. As a result, the likelihood of motivating self-satisfied people to change is very low. As for PA, thin girls (UW based on BMI) were the least active among girls, while the least active male groups were the extremes: thin boys (UW) and fat boys (OW) or those who perceived themselves as fat. Probably this pattern was due to the reason that they perceived their appearance as satisfactory and PA as a tool for weight reduction. According to regression analyses, BMI, sex, and age accounted for 30% of the variance in participants’ body perception; BMI, sex, and age accounted for 22% of body satisfaction variance, and PA was not related to either body perception or body satisfaction. PA as a variable does not explain the BI of Israeli adolescents.	13
Cheah et al., 2014 [36] -cross-sectional-	Malaysia	M: 49 F: 96	13–15	*Nutritional status* M Thinness and below: 4% NW: 63% OW: 14% Obese: 18% F Thinness and below: 3% NW: 73% OW: 16% Obese: 8%	*Mean PA* M: 6699.67 ± 3605.46 step/day F: 6022.55 ± 2775.66 step/day M + F: 6251.37 ± 3085.31 step/day (Pedometer clipped at the waist for 1 week)	*Body size discrepancy score* M: 0.96 ± 0.89 F: 1.29 ± 0.82 M + F: 1.18 ± 0.89 (Body size discrepancy score = difference between perceived current body size and perceived ideal body size)	9 silhouettes developed by Thompson and Gray [37]	The study found low PA and OW/obesity in nearly one-third of the respondents. The mean steps per day were lower than international standards for both genders. Girls were more aware of their bodies and concerned about their appearance while performing PA. Association of BI or BID with PA or BMI or weight status not assessed.	12
Schuster et al., 2014 [38] -longitudinal-	3 USA communities (Birmingham, AL, Houston, TX, and Los Angeles County, CA)	M: 1958 F: 2003 M + F: 3961	*Mean* Survey 1: 11.1 ± 0.5 Survey 2: 16.1 ± 0.5	*Weight status* *Survey 1* UW: 1% NW: 53% OW: 19% Obese: 26% *Survey 2* UW: 2% NW: 60% OW: 18% Obese: 20%	3.8 ± 2.0 days past week (PA measured as the number of days in the past 7 in which the child was engaged in vigorous exercise for ≥20 min)	BID 5% much heavier than ideal 29% somewhat heavier than ideal 50% same as ideal 15% thinner than ideal (BID: “Much heavier” indicates those whose self-image was >2 levels heavier than their ideal BI, “somewhat heavier” indicates those whose self-image was 1 level heavier than ideal, and “thinner” indicates those whose self-image was thinner than ideal)	7 silhouettes developed by Collins [34]	BID was related to the BMI category: obese and OW students were more likely than NW students to identify as being much heavier or somewhat heavier than ideal. Students were less likely to become obese if they performed more vigorous exercise. Association of BI or BID with PA not assessed.	14
Chongwatpol and Gates, 2016 [39] -cross-sectional-	Bangkok Metropolitan Region, Thailand	M: 1026 (700 in Single-sex schools; 326 in Mixed-sex schools) F: 1056 (497 in Single-sex schools; 559 in Mixed-sex schools)	15–18	*Mean BMI by type of school* M 21.5 ± 0.2 (Single-sex schools) 21.0 ± 0.3 (Mixed-sex schools) F 20.1 ± 0.2 (Single-sex schools) 20.9 ± 0.2 (Mixed-sex schools) *Weight status* M Very UW: 2% UW: 10% NW: 67% OW: 14% Obese: 8% F Very UW: 1% UW: 6% NW: 81% OW: 8% Obese: 4%	*Mean PA score* M + F: 2.2 ± 0.6 *PA score by type of school* M 2.3 ± 0.02 (Single-sex schools) 2.6 ± 0.04 (Mixed-sex schools) F 2.1 ± 0.03 (Single-sex schools) 2.1 ± 0.03 (Mixed-sex schools) (PAQ-A modified according to Janz et al. [40]: the total PA score is an average of eight items with a score of 1 indicating low PA and 5 high PA)	*Mean BID by type of school* M 1.16 ± 0.03 (Single-sex schools) 1.02 ± 0.04 (Mixed-sex schools) F 1.18 ± 0.04 (Single-sex schools) 1.11 ± 0.04 (Mixed-sex schools) The mean female desired body shape: 3.14 ± 0.82. No BD in 18% of females and 21% of males. Desire for a thinner figure in two-thirds of girls and 44% of the boys. Desire for a bigger figure in 35% of males.	9 silhouettes developed by Stunkard et al. [29]	More than 80% of adolescents reported being dissatisfied with their current body shape. Overall BID, when measured as an absolute value, was similar in male and female Thai adolescents but the majority of girls wanted to be thinner, while boys wanted to be either smaller or bigger. Among risk factors, lower socioeconomic status, higher BMI, and lower PA were found to be associated with higher body dissatisfaction explaining 20% of its variation. Participants from single-sex schools reported higher body dissatisfaction than those from mixed schools. In general, Thai adolescents reported a low PA.	12
Coelho et al., 2016 [41] -cross-sectional-	Portugal	M: 284 F:245 M + F: 529	10–18 *Mean* 13.32 ± 1.59	*Weight status:* M + F NW: 77% OW: 18% Obese: 4%	*Sports activity* 52% 0–1 day/week 24% 2–3 days/week 24% ≥4 days/week (question about frequency of sports activities expressed as day/week)	M: 44% satisfied 16% want to be larger 40% want to be thinner F: 39% satisfied 11% want to be larger 50% want to be thinner *Dissatisfaction by weight status:* *NW:* 50% satisfied; 16% want to be larger; 34% want to be thinner. *OW:* 18% satisfied; 4% want to be larger; 78% want to be thinner. *Obese*: 4% satisfied; 96% want to be thinner.	7 silhouettes developed by Collins [34]	A significant association was found between obesity and body dissatisfaction: obese and OW adolescents were more dissatisfied with their BI (96% and 78%, respectively) and wished to be thinner. Of the NW group, only 49.8% were satisfied with their BI. Obesity and four or more days per week of sports activities (OR = 0.52; IC95%: 0.32–0.84) were associated with BID. Sports activity was the only variable acting as a preventive factor for BID.	11
Rinaldo et al., 2016 [42] -repeated measures (after 12 weeks of training)-	Ferrara, Emilia-Romagna region, Italy	M: 36 soccer players	Age category: 10 years (The authors had also examined another group in the age category of 9 years)	Survey 1 Mean BMI: 19.4 ± 2.9 *Weight status* UW: 3% NW: 58% OW/obese: 39% Survey 2 Mean BMI: 19.6 ± 3.1 *Weight status* UW: 6% NW:56% OW/obese: 39%	4 h/week of soccer (question about the amount of soccer training)	*Mean BID* Survey 1: FID: 0.5 ± 1.1 Survey 2: FID: 0.2 ± 1.0	7 silhouettes developed by Collins [34]	The 10-year-old soccer players showed greater anthropometric changes and lower BID after 12 weeks of training than the nine-year-old boys. Boys aged 10 reported a general improvement in BI satisfaction after the training. Association of BI or BID with BMI or weight status not assessed.	11
Shaban et al., 2016 [43] -cross-sectional-	All six governorates in the State of Kuwait	F: 169	10–14	*Weight status* *UW/NW: 39%* *OW: 17%* *Obese: 44%* (Three BMI categories were constructed based on the percentiles: UW/NW: < 85th OW: 85th–94th Obese: ≥ 95th)	*Perceived PA (yes) by BMI* UW/NW: 80% OW: 56% Obese: 64% (Perceived PA was measured with a single question, “Do you consider yourself to be physically active?” with the answer: yes or no)	*BI satisfaction by BMI* UW/normal:68% OW: 30% Obese: 12% (Using 9 figures, the image was assigned to one of four categories: images 1, 2, and 3 = UW, images 4 and 5 = NW, images 6 and 7 = OW, and images 8 and 9 = obesity)	9 silhouettes developed by Stunkard et al. [29]	The level of engagement in perceived PA was not significantly associated with BID. In this study, 50% of the girls classified as obese (32%) regarded themselves as being NW. Obesity was not found to be associated with sedentary activities.	9
Michels and Amenyah, 2017 [44] -cross-sectional-	Accra, Ghana Akan 46% Ga-Adangme 25%	M + F: 370 (48% males)	11–18 *Mean* 15.5 ± 1.8	*Mean BMI* z-score: 0.09 ± 1.20 *Weight status* UW: 5% NW: 76% OW/obese: 19%	Mean MVPA 323.9 ±160.3 min/day (IPAQ to compute the total time for MVPA per day in a typical week)	*Mean discrepancy based on silhouettes* −0.3 ± 1.2 *Self-Report dissatisfaction* Body too thin: 42% Body satisfactory: 40% Body too heavy: 18%	9 silhouettes developed by Stunkard et al. [29]	Dissatisfaction with body size and ideal body size was not correlated with PA levels in the Ghanaian population. Dissatisfaction with body size in Ghanaians concerned both “too thin” and “too heavy” bodies.	12
Robbins et al., 2017 [45] -cross-sectional study including secondary analysis of baseline data from a group-randomized controlled Trial-	Midwestern U.S. Black: 50% White: 27% Hispanic: 14%	F: 1519	10–15 *Mean* 12.05 ± 1.01	*Weight status* UW: 3% NW: 45% OW: 20% Obese: 32% (UW: BMI-P < 5th NW: 5th to < 85th BMI-P OW: 85th to < 95th BMI-P Obese: ≥ 95th BMI-P)	*PA (min/h) by accelerometer* sedentary activity: 39.78 ± 18.30 light PA: 17.84 ± 3.58 moderate PA: 2.09 ± 0.80 vigorous PA:0.74 ± 0.53 *Self-reported PA* participated in the recommended 60 min of MVPA for 7 days: 9%. engaged in at least 60 min MVPA for 7 days: 7% (accelerometer and self-reported questionnaire to assess PA. Accelerometer cut-points: sedentary activity ≤ 25 counts/15 s; light PA 26–573 counts/15 s; moderate PA 574–1002 counts/15 s, and vigorous PA ≥ 1003 counts/15 s)	*Mean BID* 1.10 ± 1.48 (range: from −5 to +7) About 42% had an absolute discrepancy score >1. *BID/weight status* UW: 0.61 ± 1.99 NW: 0.35 ± 1.30 OW: 1.37 ± 1.07 Obese: 2.04 ± 1.28 *Ideal vs. current figure* Larger: 11% Similar: 22% Smaller: 67% 72% of white girls vs. 64% of black girls desired a smaller body image (BID: current/actual—ideal rating. Scores range between − 8 and − 1 for girls who want a larger figure and between 1 and 8 for those who prefer a thinner one)	9 silhouettes developed by Thompson and Gray [37]	Black girls experienced less BID than white girls. In general, higher BMI values are associated with lower body satisfaction, more negative body image, and a greater discrepancy between current and ideal BI. Negative correlations with BID were very low for MV, indicating that PA had an indirect effect on BID.	10
Min et al., 2018 [46] -longitudinal: a long-term prospective open-cohort study from the China Health National Survey 2000–2011- (We considered only the cross-sectional study of adolescents surveyed in 2011)	China	M: 787 F: 749 M + F: 1536	12–17 (the authors had also examined another age group in the range 6–11).	*Weight status* M UW: 17.3% NW: 62.8% OW: 19.9% F UW: 22.6% NW: 63.8% OW: 13.6% *Self-rated BI/actual weight status* M Consistent: 40% Underestimates: 54% Overestimates: 5% F Consistent: 45% Underestimates: 49% Overestimates: 6%	Mean values not reported. (MVPA assessed as min/day spent on six specific sports activities)	*Self-rated BI* M Thin: 56% Average: 41% Fat: 3% F Thin: 58% Average: 41% Fat: 1% *Desired BI* M Thin: 41% Average: 59% Fat: 0.4% F Thin: 52% Average: 47% Fat: 0% *Discrepancy between self* vs. *desired BI* *M:* 65% Consistent; 12% Need to lose weight; 24% Need to gain weight. *F:* 70% Consistent; 13% Need to lose weight; 17% Need to gain weight. (For adolescents’ self-ratings, the silhouettes were grouped into three levels: thin (silhouettes 1–3), average (silhouettes 4–6), and fat (silhouettes 7–9).)	9 silhouettes Matching Task for adolescents aged 12–17 (given by the authors in appendix 1 of their article)	Most of the adolescents perceived themselves as thin, and only 2% considered themselves fat. The least consistency between BI and actual weight (boys: 14%, girls: 5%) occurred in OW adolescents, while UW had the greatest consistency (boys: 72%, girls: 76%). Boys were more likely to desire an average body than girls, which preferred thinner bodies, regardless of their weight status. Boys and girls who needed weight loss were more likely to try dieting and less likely to feel they had enough/lots of PA than others. The discrepancy between BI and desired BI results in significant differences in weight control efforts and insufficient PA awareness in Chinese children. Associations between BI and health behaviors and risk of obesity were found.	11
Miranda et al., 2018 [47] -cross-sectional-	Viçosa, Minas Gerais, Brazil	F: 120	14–19 *Mean* 16.5 ± 1.5	*Weight status:* NW:78% OW/obese: 21% *Body satisfaction/weight status* Eutrophic/low weight: 49% satisfied; 31% dissatisfied. OW/obese: 4% satisfied; 16% dissatisfied.	*PA level* Sedentary/Low PA: 84% Active: 14% Very active: 1% *Body satisfaction vs. PA:* Active/very active: 4% satisfied; 12% dissatisfied. Sedentary/low PA: 50% satisfied; 34% dissatisfied. (the 24-hour Physical Activity Recall (24 h-PAR))	*BID* Satisfied: 50% Dissatisfied: 50% (29% wishing for a slimmer silhouette and 20% for a thicker silhouette). *Body distortion*: 51% (26% seeing as bigger; 26% seeing as smaller) (Body satisfaction was present when variation between current and ideal figures was between −1 and +1. When the difference was > +1: desire for a bigger silhouette. When the difference was < −1: desire for a smaller silhouette. Body distortion = difference between the BMI of the silhouette chosen as current and the actual measured BMI)	15 silhouettes developed by Kakeshita et al. [48] for the adult and validated by Laus et al. [49]	Nearly half of the sample showed dissatisfaction with their weight and BI. Physically active adolescents had higher BID in comparison with sedentary or inactive girls. Body composition measures (including BMI), along with sedentary behavior and PA level, were found to be correlated with BID in adolescent girls.	13
Fernández-Bustos et al., 2019 [50] -cross-sectional-	La Roda, Spain	M: 284 F: 350 M + F: 634	12–17 *Mean* 14.57 ± 1.51	*Mean BMI* M: 22.36 ± 3.93 F: 21.81 ± 3.64 M + F: 22.05 ± 3.78 *Weight status* M UW: 3% NW: 59% OW: 28% Obese: 9% F UW: 5% NW: 67% OW: 23% Obese: 5% M + F UW: 4% NW: 64% OW: 25% Obese: 7%	*MVPA (min/week)* M 281.5 ± 203.2 F 90.5 ± 137.1 M + F 176.0 ± 194.6 *PA practice* *M:* 24% inactive; 27% MVPA < 60 min/day; 50% MVPA ≥ 60 min/day. *F:* 63% inactive; 28% MVPA <60 min/day; 9% MVPA ≥60 min/day. *PA type* *M:* 59% non-aesthetic/non-lean; 27% non-aesthetic/lean; 14% aesthetic/lean. *F:* 36% non-aesthetic/non-lean; 18% non-aesthetic/lean; 46% aesthetic/lean. (IPAQ-SF)	*Mean BID* M *DDPB:* 0.60 ± 2.21 F *DDPB:* 2.21 ± 2.61 M + F *DDPB:* 1.49 ± 2.56 (DDPB = Discrepancy Desired—Perceived Body. It is based on: central figure = 0; negative values to figures to its left; positive values to figures to its right.)	13 silhouettes developed by Gardner et al. [51] adapted for Spanish by Rodríguez et al. [52]	Sex and BMI are crucial variables for BID in adolescents and PA type was a determinant variable for BI perception for both genders, regardless of BMI. Males showed lower levels of dissatisfaction. The boys showed that MVPA was moderately associated with higher BID and PA participation and organization. In girls, BI concerns were significantly lower among those who practiced non-aesthetic/light sports than among those who practiced aesthetic/light PA or were inactive. The study showed that BID does not depend on the organization or level of competition but on the type of PA practice.	11
Zaccagni et al., 2019 [53] -cross-sectional-	Emilia-Romagna region, Italy	F CRG: 36 CG: 71 (an examined group of younger non-competitive gymnasts was not reported)	*Mean* 10.7 ± 1.6	*Mean BMI* CRG: 16.9 ± 1.8 CG: 11.4 ± 0.3 (CRG: competitive rhythmic gymnasts CG: control group)	13.1 ± 4.9 h/week of training (question about the amount of weekly training for gymnasts)	*Mean BID* *CRG:* FID: 0.4 ± 0.9 FIDSport: 1.1± 1.1 *CRG:* 45% satisfied (FID = 0); 45% want to be thinner (FID > 0);10% want to be larger (FID < 0). *CG* 40% satisfied (FID = 0); 50% want to be thinner (FID > 0); 10% want to be larger (FID < 0). (FIDSport: feel figure—ideal figure in the sport)	7 silhouettes developed by Collins [34]	Both female athletes and nonathletes desired to have a leaner BI. The ideal BI of competitive girls in gymnastics was found to be thinner, confirming that the ideal figure in sports does not coincide with the ideal figure in daily life. In aesthetic sports, such as rhythmic gymnastics, it is important to assess the “sport” body image dissatisfaction rather than general BID. The practice of rhythmic gymnastics and BMI were negatively correlated with the general ideal figure.	11
Boraita et al., 2020 [54]; Boraita et al., 2022 [55] -cross-sectional-	La Rioja, Spain	M: 383 F: 378 M + F:761	12–17 *Mean* 14.51 ± 1.63	*Mean BMI* M: 21.01 ± 3.26 F: 21.02 ± 3.26 *Weight status* M NW: 72% OW: 20% Obese: 9% F NW:76% OW: 19% Obese: 5%	*Mean PA score* M: 2.74 ±0.61 F: 2.46 ± 0.59 *PA tertiles by weight status:* *Low tertile PA:* NW 31% OW/obesity *40*% *Medium tertile PA*: NW 35%; OW/obesity 28% *High tertile PA*: NW 33% OW/obesity 31% (PAQ-A)	*Desire for BMI change* M –a lower BMI: 36% –a higher BMI: 23% F –a lower BMI: 50% –a higher BMI: 12% *Dissatisfaction vs. PA* M + F *Low PA* dissatisfied: 38%; satisfied: 27%. *Medium PA* dissatisfied: 33%; satisfied:34%. *High PA* dissatisfied: 29%; satisfied: 39%	9 silhouettes developed by Stunkard and Stellar [32] adapted by Marrodàn et al. [56]	Low engagement in PA was found to be predicted by higher age, female sex, lower to middle SES, environments unfavorable to PA, non-practice of extracurricular sports, and BID. Lower levels of PA were found in students with OW/obesity and dissatisfaction with their BI. In the general sample, no significant correlation was found between PA level and BID in both genders.	13 16
Tebar et al., 2020 [57] -cross-sectional-	Presidente Prudente, Brazil	M: 482 F: 592 M + F: 1074	10–17 *Mean* M: 12.9 ± 2.4 F: 13.4 ± 2.3 M + F: 13.1 ± 3.5	*Mean BMI* M: 20.3 ± 4.3 F: 20.5 ± 4.3	*PA score* M: 9.5 ± 2.7 F: 8.8 ± 2.8 (PA was assessed by the Baecke questionnaire. The sum of the scores of three domains indicates the total score of habitual PA with a range from 3 to 15)	*BMI vs. BID* Satisfied: 18.7 ± 2.7 kg/m^2^ Desire to increase: 18.0 ± 3.2 kg/m^2^ Desire to decrease: 22.5 ± 4.3 kg/m^2^ *PA score vs. BID* Satisfied: 9.3 ± 2.7 Desire to increase: 8.9 ± 2.6 Desire to decrease: 9.1 ± 2.7 *Dissatisfaction with OW/obesity* Satisfied: 13% Desire to increase: 7% Desire to decrease: 47% (BID: difference between the perceived and desired silhouette)	11 silhouettes developed for children by Kakeshita et al. for the Brazilian Silhouettes’ Scale [48]	Dissatisfaction with body size was found in 77% of the sample. No significant sex differences were observed in BID. No association between specific PA domains (school, sports, and leisure) and dissatisfaction with body size was found. BID is considered a self-perceived obstacle to PA. Dissatisfaction with body size has been associated with OW, unhealthy eating patterns, and lower levels of PA in Brazilian adolescents.	15
Miranda et al., 2021 [58] -cross-sectional	Viçosa, Minas Gerais, Brazil	F: 405	14–19 *Mean* 15.92 ± 1.27	Mean BMI = 21.73	*PA level* Inactive: 83% MVPA < 60 min/day: 41.5% (Digiwalker SW 200 Pedometer in a 24 h period. A cut-off value of 11,700 steps to distinguish an active or inactive behavior. The 24 h PA Recall complemented this evaluation)	*BID evaluation* Dissatisfied: 51% *Body distortion:* 53% (Body satisfaction was assessed by the difference between ideal and current silhouettes: satisfaction when the difference is between –1 and +1. Body distortion = difference between the BMI of the silhouette chosen as current and the actual measured BMI)	15 silhouettes validated by Laus et al. [49] (Although unspecified, these were probably the silhouettes developed by Kakeshita et al. [48] for the adults)	More than half of the participants were dissatisfied and had a distortion of their BI. Girls with an “inactive and sedentary” latent lifestyle were 1.71 times more likely to feel dissatisfied than those with an active and sedentary or inactive and non-sedentary lifestyle. BI disorders were associated with a decreased amount of MVPA.	13
Niswah et al., 2021 [59] -cross-sectional-	Klaten and Lombok Barat districts, Indonesia	M: 1077 F: 1067	12–18	*Nutritional status* M Stunting: 19% Thinness: 10% OW/obese: 14% F Stunting: 23% Thinness: 4% OW/obese: 11% (Indicators: Stunting = height-for-age z-score < −2SD thinness = BMI for age z-score < −2 OW/obesity = BMI for age z-score >1 according to 2007 WHO growth	*Compliance with daily recommendations for PA* M: 37% F: 35% (PA recommendation (WHO): 12–17 years: at least 60 min of MVPA daily) (7-day activities frequency table to assess the number of days of activity performed and the average duration of the performance of each activity)	*BI perception* M Thin: 59% NW: 28% OW/obese: 13% F Thin: 60% NW: 27% OW/obese: 13% *BI perception/nutritional status* M *Thin status:* 94% perceived thin; 5% NW; 1% OW/obese. *OW/obese status:* 6% perceived thin; 24% NW; 70% OW/obese. F *Thin status:* 92% perceived thin; 7% NW; 1% OW/obese. *OW/obese status:* 8% perceived thin; 25% NW; 67% OW/obese.	9 silhouettes from the Figure Ranking Scale (Although unspecified, these are probably the silhouettes developed by Stunkard et al. [29])	In Indonesia 1 in 4 adolescents felt pressured to achieve the IBI indicated as thin for girls and muscular for boys. More girls than boys were dissatisfied with their body size and shape and more boys than girls felt neutral or satisfied with their BI. More OW/obese adolescents were reported to be dissatisfied with their BI than their thin counterparts. Despite strong weight-loss intentions, OW adolescent girls who perceived themselves as such did not enhance their eating or PA behaviors. Perceived BI was associated with eating and PA behaviors only in OW/obese girls but not in boys.	12
Sanchez-Miguel et al., 2021 [60] -cross-sectional-	Spain	M: 1127 F: 899 M + F: 2026	12–14 *Mean* M: 13.13 ± 0.92 F: 13.06 ± 0.86	Mean BMI M: 21.12 ± 3.84 F: 21.06 ± 3.70 M + F: 21.07 ± 3.68	*PA score* M: 2.30± 0.50 F: 2.12 ± 0.47 M + F: 2.21 ± 0.50 (Spanish version of the Physical Activity Questionnaire [61] for Adolescents (PAQ-A))	*Mean BID* M: 2.52 ± 14.80 F: 3.22 ± 14.03 M + F: 2.78 ± 14.38	4 figures neutral as to sex, age, and race from the Spanish version of the BIDA questionnaire [62]	PA was negatively associated with BMI and BID; BMI and BID were positively associated. PA was significantly associated with BID among OW/obese adolescents. No sex differences were found in BID among boys and girls who were OW/obese, and sex-per PA interaction was not significant among these weight status categories. Adolescents, particularly NW boys, with higher levels of PA are less likely to be dissatisfied with their BI.	14
Toselli et al., 2021 [63] –longitudinal- (survey after one year and after 2 years)	Emilia-Romagna region, Italy	M:64 F: 70 M + F: 134	*Mean* Survey 1 M:11.8 ± 0.3 F: 11.9 ± 0.3	*Mean BMI* M Survey 1:19.53 ± 2.89 Survey 2:19.75 ± 2.61 Survey 3:20.29 ± 2.52 F Survey 1 18.85 ± 2.44 Survey 2 19.59 ± 2.46 Survey 3: 20.75 ± 2.65 *Weight status* M *Survey 1*: NW: 69%; OW: 27%; Obese: 5%. *Survey 2*: NW: 76%; OW:24%. *Survey 3*: NW: 78%; OW:22%. F *Survey 1*: UW: 1%; NW: 82%; OW:18%; Obese: 1%. *Survey 2*: UW:1%; NW:86%;OW:13%. *Survey 3*: NW: 83%; OW:17%.	*Sports practice* M Survey 1: 92% Survey 2: 92% Survey 3: 90% F Survey 1: 90% Survey 2: 87% Survey 3: 90% *Amount of sports practice* *(h/week)* M Survey 1: 3.74 ± 1.52 Survey 2: 4.53 ± 2.23 Survey 3: 4.85 ± 1.98 F Survey 1: 3.53 ± 2.45 Survey 2: 4.22 ± 2.72 Survey 3: 4.06 ± 2.53 (PA was assessed by questions on sports practice (yes, no) and amount (h/week))	*Mean BID (FID)* M Survey 1: 0.27 ± 1.45 Survey 2: 0.22 ± 0.96 Survey 3: 0.05 ± 0.79 F Survey 1: 0.54 ± 1.26 Survey 2: 0.54 ± 0.79 Survey 3: 0.54 ± 0.88	9 silhouettes from the Body Silhouette Chart (Sanchez-Villegas et al.) [64]	Associations of BI perception with BMI and sex were found. The girls showed a lower incidence of OW and obesity than boys, but girls had a higher dissatisfaction than males. Dissatisfaction and overestimation of one’s weight status increase as BMI increases to a greater extent in females than in males. Association of BI or BID with PA not assessed.	14
Vaquero-Solis et al., 2021 [65] Sanchez-Miguel et al., 2020 [66] -cross-sectional-	Spain	M:150 F:153 M + F: 303	10–13 *Mean* 11.74± 0.86	*Mean BMI* M: 18.83 ± 3.27 F: 18.52 ± 2.73 M + F: 18.60 ± 3.00	*PA score* M + F 3.20 ± 0.67 (PAQ-A)	*Mean BID (FID)* M: 0.54 ± 1.31 F: 0.51 ± 0.95 M + F: 0.52 ± 1.14	9 silhouettes developed by Stunkard et al. [29]	Significant positive relationships were shown between BMI and BID. Moreover, significant negative relationships were found between BMI and PA. Association of BI or BID with PA not assessed.	9 10
Lai et al., 2022 [67] -cross-sectional-	Seremban, Negeri Sembilan, Malaysia Malay 83%	M:1011 F: 1210	12–18	Mean BMI 21.17 ± 5.33 *Nutritional status* M Severe thinness: 2% Thinness: 7% NW:58% OW:16% Obese:17% F Severe thinness: 1% Thinness: 3% NW:64% OW:18% Obese:13%	*PA level* M Low: 48% Moderate/high: 52%. F Low: 65%. Moderate/high: 35%. M + F Low: 57% Moderate/high: 43%, (PAQ-C)	*Mean BID* M DS: 0.18 ± 1.20 F DS: 0.71 ± 1.35 M + F DS: 0.46 ± 1.31 (Discrepancy score (DS) in body size satisfaction = current − ideal)	9 silhouettes developed by Thompson and Gray [37]	OW and obese participants had significantly higher body discrepancy scores than their NW or thin counterparts. A third of the OW/obese participants underestimated their weight status, more than one-third were unaware that they were OW, and 83% of OW/obese perceived themselves to be smaller. Low levels of PA and body dissatisfaction were significant predictors of OW and obesity.	13
Leppänen et al., 2022 [68] -cross-sectional-	Helsinki, Turku, Espoo, Oulu, Jyväskylä, Tampere, Kuopio, Finland	M: 4980 F: 5516 M + F: 10,496	9–12 *Mean* M: 11.2 ± 0.84 F: 11.1 ± 0.85	*Nutritional status* M Thin: 9% NW: 75% OW/obese: 15% F Thin: 13% NW: 72% OW/obese: 15%	*PA level* M Low: 32% Moderate: 30% High: 38% F Low: 41% Moderate: 31% High: 27% (Based on a questionnaire: PA ≤ 5 h/week (low level); PA = 6–8 h/week (moderate level); PA ≥ 9 h/week (high level).)	*BID categories* M -wishing for a smaller body: 27% -satisfied: 63% -wishing for a bigger body: 10% F -wishing for a smaller body: 32% -satisfied: 59% -wishing for a bigger body: 9% (Three groups based on the difference between the wanted and current BI (wishing for a smaller body, satisfied, wishing for a bigger body))	7 silhouettes developed by Collins [34]	BI was associated with both BMI and PA. Girls had higher odds of wishing for a different body in comparison with boys. Having an NW or a high level of PA was associated with higher BI satisfaction. Adolescents with low and moderate PA levels had lower odds of wishing for a bigger body than adolescents with high PA levels. PA level modified the associations between BMI and BI, especially in thin adolescents and more in girls than in boys.	14
Song et al., 2022 [69] -cross-sectional from the China Health National Survey 2015-	China from rural (34%) and urban (66%) areas	M: 729 F: 640 M + F: 1369	6–17 *Mean* M: 10.38 ± 3.18 F: 10.37 ± 3.06 M + F: 10.37± 3.12	*Mean BMI* M: 18.08 ± 3.39 F: 17.61 ± 3.20 M + F: 17.86 ± 3.31 *Weight status* M: UW: 7% NW: 64% OW/obese:29% F UW:9% NW: 66% OW/obese:20% *Actual vs. self-perceptive weight status* M *UW:* 42% perceived UW; 53% perceived NW; 2% perceived OW/obese. *NW:* 17% perceived UW; 78% perceived NW; 5% perceived OW/obese. *OW/obese:* 7% perceived UW; 61% perceived NW; 32% perceived OW/obese. F *UW:* 43% perceived UW; 55% perceived NW; 2% perceived OW/obese. *NW:* 14% perceived UW; *76*% perceived NW; 9% perceived OW/obese. *OW/obese:* 8% perceived UW; 71% perceived NW; 21% perceived OW/obese.	*PA practice* M No: 59% Regular: 40% F No: 63% Regular: 36% M + F No: 61% Regular: 38% (Information on PA (no, regularly) through CHNS (2015) questionnaires)	*BID* M Satisfied: 40% Desire to be heavier: 36% Desire to be thinner: 23% F Satisfied: 41% Desire to be heavier: 33% Desire to be thinner: 26% (BID= present body FRS score—ideal body FRS score. Desire to be thinner: BID score ≥ 1; desire to be heavier: BID score ≤ −1; satisfaction: BID score = 0.)	9 silhouettes developed by Stunkard et al. [29]	About two-fifths of the total sample had a misperception of their weight status, and more than half of the total sample was dissatisfied with their BI. Boys tend to underestimate their weight status, while girls are more likely to overestimate it. In particular, females would be more sensitive to their weight status and BI due to external and internal pressures than males. Only the association between body perception and behaviors of dietary weight management was assessed.	14

Note: M: males; F: females; MVPA: moderate/vigorous physical activity; UW: underweight; NW: normal weight or healthy weight; OW: overweight; BI= body image; IBI: ideal body image; BID: body image dissatisfaction; FRS: figure rating scale; FID: feel minus ideal discrepancy. Underlined and italicized text indicates data calculated by us.

## Data Availability

Not applicable.

## Supplementary Materials

The following supporting information can be downloaded at: https://www.mdpi.com/article/10.3390/ijerph192013190/s1; Table S1: Prisma 2020 Checklist; Table S2: NOS scores for all included studies in alphabetical order (range: 0–16 stars, with higher scores indicating better quality research). The range of each item was reported.
